# Perspectives for Forkhead box transcription factors in diabetic cardiomyopathy: Their therapeutic potential and possible effects of salvianolic acids

**DOI:** 10.3389/fcvm.2022.951597

**Published:** 2022-08-11

**Authors:** Ronghui Han, Hemeng Huang, Weiyi Xia, Jingjin Liu, Hui Luo, Jing Tang, Zhengyuan Xia

**Affiliations:** ^1^Department of Anesthesiology, Affiliated Hospital of Guangdong Medical University, Zhanjiang, China; ^2^Department of Emergency, Affiliated Hospital of Guangdong Medical University, Zhanjiang, China; ^3^Department of Orthopaedics and Traumatology, The Univerisity of Hong Kong, Hong Kong, China; ^4^Department of Cardiology, Shenzhen People’s Hospital and The First Affiliated Hospital, Southern University of Science and Technology, Shenzhen, China; ^5^Marine Biomedical Research Institution, Guangdong Medical University, Zhanjiang, China; ^6^State Key Laboratory of Pharmaceutical Biotechnology, Department of Medicine, The University of Hong Kong, Hong Kong, China

**Keywords:** diabetic cardiomyopathy (DCM), the class O of Forkhead box (FoxO) transcription factors, FoxO1, FoxO3a, salvianolic acid

## Abstract

Diabetic cardiomyopathy (DCM) is the primary cause of morbidity and mortality in diabetic cardiovascular complications, which initially manifests as cardiac hypertrophy, myocardial fibrosis, dysfunctional remodeling, and diastolic dysfunction, followed by systolic dysfunction, and eventually end with acute heart failure. Molecular mechanisms underlying these pathological changes in diabetic hearts are complicated and multifactorial, including but not limited to insulin resistance, oxidative stress, lipotoxicity, cardiomyocytes apoptosis or autophagy, inflammatory response, and myocardial metabolic dysfunction. With the development of molecular biology technology, accumulating evidence illustrates that members of the class O of Forkhead box (FoxO) transcription factors are vital for maintaining cardiomyocyte metabolism and cell survival, and the functions of the FoxO family proteins can be modulated by a wide variety of post-translational modifications including phosphorylation, acetylation, ubiquitination, arginine methylation, and O-glycosylation. In this review, we highlight and summarize the most recent advances in two members of the FoxO family (predominately FoxO1 and FoxO3a) that are abundantly expressed in cardiac tissue and whose levels of gene and protein expressions change as DCM progresses, with the goal of providing valuable insights into the pathogenesis of diabetic cardiovascular complications and discussing their therapeutic potential and possible effects of salvianolic acids, a natural product.

## Introduction

The improvement of people’s living standards has always been accompanied by a variety of chronic and inconspicuous diseases that exercise a negative influence on our mental and physical health and our longevity. Diabetes Mellitus is increasingly recognized as a serious, worldwide public concern, in particular, patients at a young age and low body max index (1). Though the fleeting high glucose at the very beginning is frequently neglected among patients with type 2 diabetes (T2D), continuous hyperglycemia will damage the vascular system and induce a range of pathological changes in hearts (1, 2). Whatever the origin with nature or nurture, a plethora of clinical and experimental studies have verified that cardiomyocytes impairment provoked directly by type 1 diabetes (T1D) and T2D would contribute to the occurrence and development of diabetic cardiomyopathy (DCM) (3, 4), and eventually, lead to lethal heart failure. Meanwhile, myocardial pathological changes and dysfunction in DCM are extraordinarily tangled and complicated, it involves abnormal insulin sensitivity and signaling, excessive oxidation products, continuous stimulation of inflammation, degeneration of heart tissue, uncontrollable apoptosis, necrosis and autophagy (5–8). As a result, understanding the pathogenic mechanism governing the development and progress of DCM and exploring the effective and controllable cardiac biomarkers for risk prediction is urgently needed.

Recently, mounting evidence from multiple scientific studies shows that Forkhead box O (FoxO) transcription factors, including FoxO1 (FKHR), FoxO3a (FKHRL1), FoxO4 (AFX) and FoxO6, have important functional implications in several signaling pathways refer to human health and diseases (9, 10). So far, two subtypes of FoxO (FoxO1 and FoxO3a) are known to be essential for the maintenance of cardiovascular homeostasis that are nearly expressed in all tissues (11–13). Studies from our laboratory also illustrated a pivotal role for FoxO1 or FoxO3a in cardiac metabolism. Our findings indicated that FoxO1 is a critical initiator of T1D-induced vascular remodeling through activating NOD-like receptor family protein-3 (NLRP3) inflammasome-dependent inflammation (14). Meanwhile, activation of FoxO1 in diabetic rats may promote a change in the preferred substrate from glucose to fatty acid and results in mitochondrial and cardiac dysfunction *via* stimulating pyruvate dehydrogenase kinase 4 (PDK4) and carnitine palmitoyltransferase 1 (CPT1) (15). Furthermore, we found that up-regulation of FoxO1 or FoxO3a is attributable to the reduction of H9c2 cardiac cells apoptosis and autophagy induced by hypoxia/reoxygenation injury under hyperglycemia *in vitro* (16). Interestingly, our findings regarding the impact of FoxO1 and FoxO3a on apoptosis and autophagy (16) are in contrast to findings of previous studies conducted in different experimental settings which showed that enhancement of cardiac FoxO1 protein expression is responsible for the increased autophagy and apoptosis in mice with DCM (17), while increase in FoxO3a activation has been shown to be a mechanism by which high glucose induced oxidative stress and apoptosis in cardiac microvascular endothelial cells (18). Available evidence suggest that the FoxO family plays an important role in the development of DCM but its molecular mechanism remains largely unclear.

This review focuses primarily on the regulation and outcomes of FoxO signaling which should be comprehensively investigated to develop novel therapeutics and preventives for DCM.

## Structure of Forkhead box O transcription factors

Human Forkhead-box gene family consists of 19 subfamilies from FoxA to FoxS and there are more than 40 members that have been identified till now (19, 20). It is noticeable that mammalians express four members FoxO1, FoxO3a, FoxO4, and FoxO6 in FoxO transcription factors (21). From the embryo to the adult, FoxO1 and FoxO3a are critical for maintaining cellular homeostasis and genetic integrity of which we will discuss below.

### Structure and general characteristics of Forkhead box O proteins

FoxO transcription factors are characterized by four domains, including an extremely conserved DNA-binding Forkhead or wing-helix domain (DBD, consists of W1/W2 wing-like loops, H1/H2/H3 α*-*helices, and S1/S2/S3 β-strands), a nuclear localization sequence (NLS, the precise amino-acid sequence in the adjacent neighborhood of the DBD for their nuclear translocation), a nuclear export sequence (NES, the reverse function of a nuclear import signaling, which targets proteins out of the cell nucleus), and a C-terminal containing a transactivation domain (TAD) as being demonstrated in previous publications (10, 22).

### Main post-translation modifications

**FIGURE 1:**
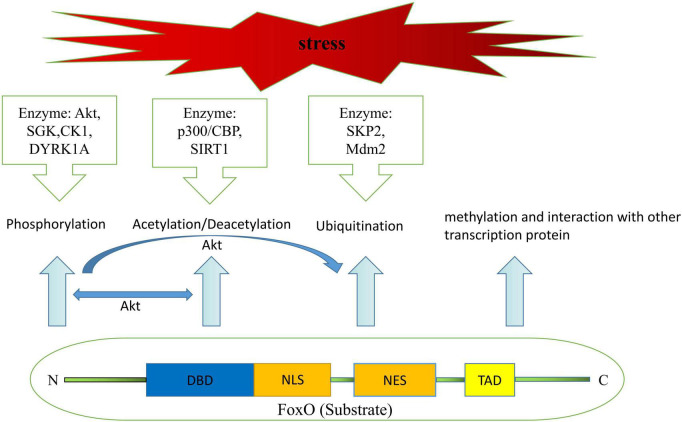
Structural components of FoxO protein and mechanisms leading to post-translational modifications of FoxO.

It is well known that regulation of transactivation domain or activation domain of FoxO transcription factors depends on post-translational modification, containing but not limited to phosphorylation, acetylation/deacetylation, ubiquitination, methylation, and interaction with other transcription protein (23) (Figure 1). Meanwhile, attenuation or enhancement of transcription function in FoxO transcription factors achieved by the above-mentioned post-translational modifications is depend on the magnitude of nuclear/cytoplasmic localization and variation of DNA recognition under some specific physiological and pathological conditions, such as fasting diet, diabetes, sepsis, ischemia and so on, and thereby trigger cellular proliferation, autophagy, apoptosis, self-degradation, etc. (24, 25).

#### Phosphorylation

Akt-dependent phosphorylation has been thought of as a key factor in cellular proliferation and survival by inhibiting the transcriptional functions of FoxO over the past two decades, as this post-translational modification would facilitate FoxO translocation from the nuclear to the cytoplasm and results in the disruption of NLS and DBD function (26–28). Emerging evidence also suggests that FoxO transcription factors could be phosphorylated by other kinases, including serum and glucocorticoid-regulated protein kinase (SGK), casein kinase-1 (CK1), dual-specificity tyrosine-phosphorylated and regulated kinase-1A (29, 30). Interestingly, Chen et al. reported that both SGK and Akt kinase have great effects on lifespan, stress resistance, and FoxO transcription factor activity in *Caenorhabditis elegans* through completely different mechanisms (31). For instance, whereas Akt-1 and Akt-2 weaken lifespan extension by secluding DAF16/FoxO in the cytoplasm (32), SGK promotes longevity in a DAF16/FoxO-dependent manner as well (30). Despite that there was a broad consensus on the fact that SGK and Akt share similar primary structure and substrate specificity, and both of them form a protein complex regulated by the 3-phosphoinositide-dependent protein kinase-1 (PDK1) or phosphatidylinositide3-kinases (PI3K) are capable of phosphorylating DAF16/FoxO (33–35). However, it is not known whether one could substitute for the other in regulating FoxO transcription factors.

#### Acetylation/Deacetylation

FoxO transcriptional activity is also modulated by acetylation/deacetylation. As more acetylation sites have been discovered, mostly in the W2 region of the DNA-binding domain, mounting evidence has described that the effects of FoxO acetylation/deacetylation are associated with two types of proteins namely histone deacetylases and histone actyltransferase which initiate fundamentally distinct feedback signals during various biological processes (36, 37). Early studies found that acetylation of FoxO factors by calcium response element-binding (CREB)-binding protein (CBP) and/or p300 increased nuclear export, which was made feasible by phosphorylation, and that this effect could be reversed by over-expression of the NAD-dependent protein deacetylase silent information regulator (SIRT1) (38–40). Deacetylation, however, has also been shown to influence FoxO activity in a dual manner (41). On the one hand, in response to stress and nutritional restriction, SIRT1 stimulates catabolic gene expression under fasting condition *via* deacetylating the Forkhead factor FoxO (42, 43). Meanwhile, SIRT1 deacetylates FoxO, thus increasing FoxO’s ability to prevent oxidative stress and inflammation (44, 45). But on the other, deacetylation promotes FoxO-induced cell-cycle arrest (41, 46). These findings pave the way to a better understanding of the processing of FoxO acetylation/deacetylation. From metabolism to longevity and open new perspectives for therapeutic targets to treat maladaptive processing of pathophysiology.

#### Ubiquitination

Excessive activation of FoxO transactivation activity disturbs the metabolic equilibrium *in vivo*. Scientists have revealed that there exists a way to ameliorate abrupt FoxO-toxicity, namely ubiquitination (29, 47). Until now, researchers believed that this post-translational modification, poly-ubiquitination-dependent proteasomal degradation, is regulated by PI3K/Akt-mediated phosphorylation and activated by S-phase kinase-associated protein-2 (SKP2) binding to FoxO at Ser256 site in the cytoplasm, but not in the nuclear (23, 48). Besides the canonical pathway of poly-ubiquitination, the mono-ubiquitination, Murine double minute-2 (Mdm2) has been identified as a novel ubiquitin E3 ligase which induces mono-ubiquitination with increased FoxO transcriptional activity *via* promoting its nuclear localization, and, it suggested an analogical model of regulation between FoxO transcription factors and p53 as well (49). It also has been validated that Mdm2 is required for extracellular signal-regulated kinase (ERK)-induced phosphorylation of FoxO3a and may function as an ubiquitin ligase for the further degradation of FoxO3a (50). Interestingly, FoxO3a protein also exhibits a poly-ubiquitination pattern in degradation that is associated with Mdm2 as mentioned in the research by Wang et al. (42). That is to say, both poly and mono ubiquitination could be switched or interconverted by Mdm2. Furthermore, neither type of ubiquitination is far from controlled, and USP7 is reported to remove ubiquitin from FoxO, and serves as a deubiquitinating enzyme (51).

## Advance in diabetic cardiomyopathy

**FIGURE 2:**
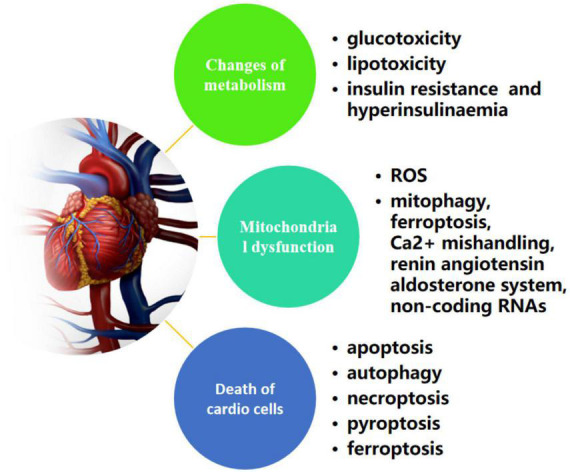
Possible mechanisms of metabolism, cell death, and mitochondrial dysfunction in diabetic cardiomyopathy.

In 1972, Rubler and his co-workers initially described the existence of a DCM based on postmortem pathological findings of four adult diabetic patients who suffered from heart failure without apparent evidence of classic clinical manifestations, such as hypertension, coronary artery disease, congenital heart defect or excessive drinking (52). Until now, multiple researches have uncovered a far-ranging potential mechanisms that attribute to the process of deterioration of DCM to heart failure, which are related to changes of metabolism (hyperglycemia, insulin resistance/hyperinsulinaemia, and dyslipidemia), various forms of programmed active cell death (apoptosis, pyroptosis, autophagic cell death, necroptosis, and ferroptosis) and mitochondrial dysfunction (53) (Figure 2). In other words, fully understanding of those possible pathogenesis is beneficial to develop pharmacological strategies combating DCM. The following sections provide an update on the current understanding of the pathophysiology of DCM.

### Changes of metabolism

Metabolic disturbance including high glucose, insulin resistance and activation of fatty acid which all are attributable to adverse myocardial structural remodeling in diabetes and often lead to cellular malfunction, and early interventions such as drugs that low blood sugar and/or reduce triglyceride levels in diabetic dyslipidemia would be beneficial in preventing or attenuating cardiac remodeling in diabetes (54).

The healthy heart can always remain delicately equilibrium to utilize myocardial energy substrates, however, this common balance would be destroyed in patients who endured unremitting high glucose levels, a situation termed as “glucotoxicity.” Both animal and cellular studies have demonstrated that glucose overload in cardiomyocytes promotes the malformation of morphology and cardiac dysfunction, for instance, hypertrophy, fibrosis, systolic or diastolic dysfunction and other pathophysiological changes (55), which can be achieved through direct or indirect activation of oxidative stress, accumulation of advanced glycation end-products (AGEs), maladaptive hexosamine biosynthesis and polyol pathway (56). Interestingly, even though epidemiological evidence has shown that glycemic control is an essential part to reduce diabetic complications (57), a phenomenon called “hyperglycemic memory” suggested that hyperglycemic stress persists in the cardiovascular system of patients with diabetes regardless of the level of blood sugar is strictly controlled (58).

In the uncontrolled diabetic state, free fatty acids oxidation nearly become the major energy resource to produce enough adenosine triphosphate (ATP) that satisfies the high demand of the heart following the distinct limitation of glucose oxidation (59). It is also noticeable that excess free fatty acids and other lipids (triglycerides, etc.) could result in lipotoxicity when metabolic disorder occurs in cardiomyocytes (60). While cardiomyocytes use fatty acids almost exclusively to support ATP synthesis, the process will consume more oxygen than using glucose for energy generation, and thus it is less efficient to sustain cardiac work as a consequence of increased production of reactive oxygen species (ROS) and accumulation of lipid intermediates such as diacylglycerols and ceramide (61–63).

The metabolic shift toward fatty acids oxidation rather than glucose oxidation is partially due to the insulin resistance or hyperinsulinaemia (64, 65). In the normal heart, 60–80% of cardiac energy provided for myocardial metabolism is derived from fatty acid oxidation, and the remaining 20–40% is derived from glucose and lactic acid metabolism (66). The utilization of glucose is further cut down when cardiac energy relies more on fatty acids oxidation under insulin resistance condition or hyperinsulinaemic state (67). And beyond that, numerous studies have reported that impaired myocardial insulin signaling under insulin resistance or hyperinsulinaemia are independently linked with the development of DCM and cardiac diastolic dysfunction through a variety of mechanisms (6, 68). For instance, Mellor et al. showed that cardiomyocytes from Glut-4-knockout mice demonstrated extreme glucose uptake deficiency and exhibited cardiac hypertrophy and marked excitation-contraction coupling abnormalities (69). On the other hand, cardiac energy deprivation due to reduction in fatty acid uptake and inhibition of ATP generation resulted from impaired FoxO1/CD36 signaling may accelerate the occurrence of concentric hypertrophy and myocardial fibrosis, exacerbating the progression of DCM (70).

### Mitochondrial dysfunction

Studies have verified the existence of defects of mitochondrial function in animal models of diabetes, and the potential regulations in mitochondrial biology in diabetes have been consistently unmasked (71–73). In particular, distinct pathways and mechanisms governing the development of mitochondrial dysfunction in DCM have been proposed in recent years (74, 75). Under normal circumstance, the energy needed to support heart function is mainly derived from free fatty acids oxidation but glucose oxidation also contribute significantly to heart energy generation. Given that fatty acid oxidation yields fewer ATP production per mole of oxygen than that from glucose oxidation, excessive fatty acid oxidant in the circumstance of impaired glucose oxidation in diabetes causes mitochondrial swelling and uncoupling due in part to the excessive consumption of molecule oxygen and the concomitantly increased production of ROS (76, 77). The damage caused by accumulation of ROS-mediated mitochondrial malfunction is not limited to intensive lipotoxicity induced by fatty acid oxidation or impaired adiponectin receptor 1 signaling, but can also be related to mitophagy, ferroptosis, Ca^2+^ mishandling, renin angiotensin aldosterone system, non-coding RNAs, and so forth (78–80). Initially, researchers observed the significant mitochondrial damage mediated by ROS-induced alteration of topoisomerase activity under chronically elevated glucose concentration in rat cardiomyocytes (81). Afterward, further studies revealed that mitophagy resulted from burst of ROS might be a compensation strategy for inconsistent autophagy flux in T2D models while the diminished autophagy appears to be an adaptive response that protects against cardiac injury in type 1 diabetic animal models (62, 82, 83). Indeed, numerous studies have demonstrated that various ROS scavengers or antioxidants are able to reduce cardiomyocyte death *in vitro* and cardiac injury *in vivo via* eliminating impaired mitochondria and protecting of the remaining intact mitochondria, however, these studies were still restricted to diabetic experiments in animals (84), and effects of treatment with various antioxidants remain largely disappointing in clinical trials (85) despite marginal treatment effects has been reported showing that alpha-lipoic acid may have a role in preventing the development of DCM in type 1 diabetes with sub-clinical left ventricular dysfunction (86).

### Death of cardiomyocytes in diabetes

In both T1D and T2D, it is widely accepted that there are 3 pivotal forms of cell death in cardiomyocytes including apoptosis, autophagy and necrosis, while necrosis can be further classified as necroptosis, pyroptosis, and ferroptosis (87, 88). From the mass of evidence available, the effective interference linked with a certain degree of suppression or activation in cardiomyocyte death of any forms yields tremendous protective consequences (89, 90).

There are two important apoptosis pathways that have been extensively studied in animal experiments including intrinsic apoptotic pathway (also known as death receptor pathway) and extrinsic pathway (called mitochondrial apoptotic program). For the intrinsic pathway, researchers confirmed that the cytochrome c-activated caspase-3 pathway caused myocardial apoptosis triggered by ROS derived from high levels of glucose in H9c2 cardiac myoblast cells (91). Meanwhile, it has been shown that galangin (Gal) attenuated cardiac apoptosis through inhibiting caspase activity in diabetic rats (92). Angiotensin II (Ang II)-dependent process is another mechanism implicated in the induction of cardiomyocyte apoptosis in diabetic rat hearts (93), and the subsequent evidence that myocardial cell apoptosis induced by Ang II could be suppressed by the application of metallothionein confirmed the involvement of the above mechanism (94). During the past few years, various non-coding RNAs (such as microRNAs and long non-coding RNAs) and exosomes are emerging as potential key regulators of apoptosis pathway in diabetes (95–98), but the specific mechanism remains incompletely elucidated. However, it is worth noting that all these complicated signaling pathways in diabetes-induced cell death seem to point to the formation of excessive ROS in the diabetic hearts.

In the heart, autophagy is important to maintain homeostasis *via* eliminating impaired or useless organelles, and thus, disruption in this pathway leads to uncontrolled and untargeted cellular suicide. However, the real role of autophagy in DCM is quite controversial. Qiao et al. found that activating the RIPK1-RIPK3 pathway by impairing the autophagic flux which increased the expression of autophagic related proteins such as LC3-II, p62, and active-cathepsin D eventually leads to myocardial fibrosis and cardiac dysfunction in diabetic rats (99). On the other hand, selective autophagic removal of mitochondria (mitophagy) appears to be an adaptive response through clearing away defective mitochondria that protects against cardiac injury in type 1 diabetes (100, 101). By contrast, other studies suggested that cardiomyocyte apoptosis may suppress autophagy in diabetic hearts (102, 103), that is to say that mutual effects between the autophagy and apoptotic cell death pathways are crucial in the pathological progress of DCM. Therefore, the impact of diabetes on cardiac autophagy and its consequences still need to be further investigated.

Necroptosis is involved in the pathogenesis of many diseases, but its role in DCM is still not clear as the relevant investigation is quite rare in both animal models or in *in vitro* studies. Some studies confirmed that myocardial cell necroptosis is observed in the hearts of diabetic patients and animal models (90, 104). Recent studies described that activation of Ca2^+^/calmodulin-dependent protein kinase (CaMKII) *via* a RIPK3-dependent manner or deficiency of sirtuin-3 (SIRT3) can exacerbate DCM through necroptosis enhancement in diabetic rats (105, 106). Even so, direct evidence for the exact mechanism of necroptosis in DCM is not very clear and more in depth investigations are merited.

There is also limited knowledge about the role of pyroptosis in DCM. As a unique and programmed form of cell death, pyroptosis has been revealed to be associated with inflammatory response in DCM *via* releasing of pro-inflammatory intracellular contents including interleukin (IL)-1β, IL-18, and other inflammatory substances that are induced subsequent to the activation of the NLRP3 inflammasome which is linked to key cardiovascular risk factors (107, 108). Meanwhile, emerging evidence has verified the role of non-coding RNAs in the pathogenesis of pyroptosis in DCM (109), including micro RNA (miRNA), circular RNA (circRNA), and long non-coding RNA (lncRNA) (110). Xu et al. proved that suppression of NLRP3 inflammasome activation-mediated pyroptosis by targeted miR-34b-3p/AHR axis from long non-coding RNA GAS5 is valuable for the attenuation of DCM (111). ROS scavengers, p38 and FoxO1 inhibitors have been shown to inhibit NLRP3 inflammasome assembly and activation in diabetic conditions and attenuate diabetic atherosclerosis through downregulating the p38-FoxO1-TXNIP (thioredoxin-interacting protein) pathway (112), and TXNIP regulates myocardial fatty acid oxidation *via* miR-33a signaling (113). Unlike previous study, another report suggested that methyltransferase-like 14 (METTL14) inhibits pyroptosis by down-regulating of lncRNA TINCR (114). Regardless of these discrepancies regarding the mechanisms of pyroptosis regulation in the context of diabetes and DCM, there is no doubt that pyroptosis may be a new therapeutic target in the prevention or treatment of DCM.

Ferroptosis is a newly described form of iron-dependent regulated necrosis that distinctly varied from apoptosis, autophagy and other types of cell death (115). Until now, numerous emerging evidences presented that ferroptosis plays an important role in the pathophysiology of cardiovascular disease including ischemia/reperfusion injury, myocardial infarction, heart failure, DCM, and cardiac arrhythmia controlled by several mechanisms, including mitochondrial activity and metabolism of iron, lipid, and amino acids (116, 117). Zang et al. substantiated that cardiac iron deposition was significantly increased in T1D rats which was promoted by activated nuclear factor erythroid2-related factor 2 (Nrf2) signaling subsequent to autophagy inhibition *via* cardiomyocyte-restricted knockout of autophagy-related 5 gene (107). Consistently, suppression of autophagy in H9C2 cardiomyocytes enhanced Nrf2-coordinated ferroptosis (118). Meanwhile, recent study described that ferroptosis was also evidenced in the heart of type 2 diabetic mice with DCM and could be inhibited by sulforaphane *via* AMPK-mediated Nrf2 activation (119). Taken together, credible and further evidences for developmental mechanism in ferroptosis of clinical patients remain blurred and the dual role of Nrf2 in the development of DCM needs to be investigated deeply.

## Regulation of Forkhead box in diabetic cardiomyopathy

**FIGURE 3:**
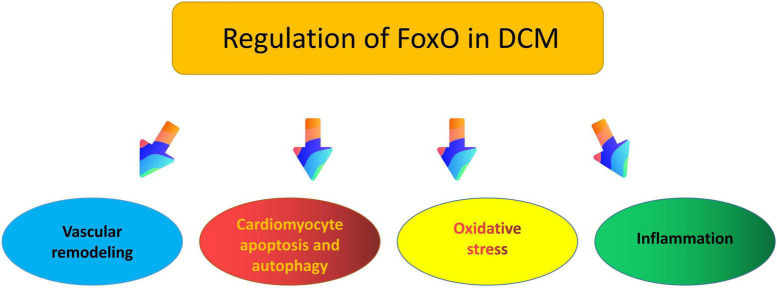
Putative roles of FoxO in the pathogenesis of diabetic cardiomyopathy.

It is well known that FoxO transcription factor plays a vital role in cardiovascular homeostasis (120), at the same time, the available evidence indicates that FoxO may impact DCM through vascular remodeling, cellular apoptosis and autophagy, oxidative stress, inflammation, and other pathophysiological processes (11, 121, 122) (Figure 3).

### Vascular remodeling

Previous study shown that FoxO1 is required for embryonic vascular development and FoxO1-null embryo died in the early stage as a consequence of incomplete vascular growth (123), while the deletion of FoxO3a may results in cardiac hypertrophy and eventual cardiac failure for the offspring (124). To elucidate the character of FoxO transcription factor, Potente et al. showed that overexpression of FoxO1 or FoxO3a led to profound inhibition of endothelial cell migration and tube formation, however, such effect is counterproductive while silencing either of FoxO1 or FoxO3a *via* using small interfering RNA (siRNA). And beyond that, researchers also further suggested that several genes with vital vascular functions are regulated by FoxO1 or FoxO3a, and gene silencing study revealed that eNOS is integral for postnatal neovascularization as a novel FoxO target gene (125). Consistent with these findings, our group first demonstrated that suppression of FoxO1 might attenuate vascular remodeling induced by type 1 diabetes in rats through decreasing the expression of NLRP3 inflammasome activation and activating related underlying mechanism including PDK1/FoxO1 pathway (14). As a result, inhibition of FoxO may provide a valuable tool to alleviate the adverse vascular remodeling in DCM but the specific and unique role of each FoxO isoform remains unclear.

### Cardiomyocyte apoptosis and autophagy

Appropriate programmed cell death such as apoptosis and autophagy seems to be an essential part in the elimination of cytotoxic proteins and dysfunctional organelles which would otherwise accelerate the development of DCM and the deterioration of DCM to cardiac failure. And, more and more evidences revealed that suppression of excessive cardiomyocyte death of aforementioned death modes occurred in the diabetic heart may yield tremendous advantageous effects on DCM (126–128). It is well known that FoxO1 and/or FoxO3a participate in the regulation of various cell death seen during insulin resistance and diabetes. With apoptosis, FoxO transcription factors are downstream targets of the Akt kinase which regulates processes of cellular proliferation and survival and also may indirectly stimulate expression of death receptor ligands such as Fas ligand through the unstable mitochondrial channels or directly induce the expression of multiple pro-apoptotic proteins like Bcl-2 family (18, 25, 129). As for autophagy, researchers have shown that plenty of the regulation and control mechanisms include but are not limited to the mTOR/AMPK, FoxOs, SIRTs, and others signalings, and further studies to unmask the detailed roles and in particular the potentially complicated interplays of the pathways should help to elucidate the inconsistent outcomes observed following the intervention of the individual pathways in the treatment of DCM (127, 130). Convincing evidences showed that certain drugs protected pancreatic β-cells by enhancing autophagy such as liraglutide (a long-acting human glucagon-like peptide-1 analog), which protected insulinoma cells *via* alleviating apoptosis accompanied by a significant increase of autophagy under hyperglycemia *in vitro* (131). And, Guo et al. proved that the improvement of islet function by liraglutide involves FoxO signaling pathway through modulating miRNA expression profiling (132). However, a recent study suggests that inadequate autophagy with impaired autophagosome-lysosomal fusion exists in the aortic intima and endothelial cells from diabetic patients and that FoxO1 may inhibit autophagosome-lysosome fusion and lead to endothelial autophagic-apoptosis in diabetes (133). In a previous study, we revealed that propofol postconditioning could attenuate hypoxia/reoxygenation-induced apoptosis and autophagy by regulating the expression of FoxO1 and FoxO3a in H9c2 cell under hyperglycemia (16). Of note, FoxO emerges as a potential participant in regulating cell death, but its role under conditions of insulin resistance and diabetes still need to be further explored.

### Oxidative stress

It is indisputable that oxidative stress plays a vital role in cardiac remodeling and malfunction, and reverse of excessive burst production of ROS by antioxidants contributes to the improvement of cardiac metabolism and function in the heart of experimental diabetic models (134, 135). Our recent study demonstrated that activation of FoxO1 *via* stimulating the expression of PDK4 and CPT led to imbalanced oxidative metabolism which may exacerbate the progress of DCM in streptozotocin-induced type 1 diabetes in rats and such disadvantageous effects like mitochondrial and cardiac dysfunction is reversible by administration of AS1842856 (AS), a selective FoxO1-selective inhibitor (15). Interestingly, we also found that up-regulation of FoxO1 or FoxO3a decreased the ROS level in H9c2 cells, a cardiac cell strain derived from the S-D Rat left ventricle, under the condition of hyperglycemia (16). In addition, researchers demonstrated that propofol could up-regulate FoxO1 to attenuate myocardial cell injury after oxygen and glucose deprivation and reperfusion (136), and Peng et al. showed that FoxO3a silencing strengthened ROS accumulation (18), these data are in agreement with our experimental findings *in vitro* but contradictory to other finding *in vivo* (137). These inconsistent results implied that oxidative stress regulates FoxO activity through various post-translational modifications including phosphorylation, acetylation, ubiquitination and so on, which may be restricted to the experimental environment as well (138). For example, inhibition of SIRT1 by Exendin-4 down-regulated the phosphorylation of FoxO1 and reduced post-ischemic infarct size and preserved the function and structure of the left ventricles in rats through scavenging free radicals directly (139), and enhancement of H4 acetylation of the FoxO3a promoter region has been shown to confer myocardium protection from oxidative stress-mediated ischemia/reperfusion injury (140). Therefore, understanding the complicated and changeable mechanisms in redox regulation of FoxO signaling is essential for the initiation of interventions that may help to maintain cardiac function in diabetes.

### Inflammation

Inflammation is significantly involved in the progression of DCM, and chronic inflammation eventually leads to decreased insulin sensitivity and diabetic complications. Hyperglycemia-induced FoxO over-activation is central to the production of pro-inflammatory cytokines.

Lundell et al. showed that several inflammatory signaling pathways are enriched by FoxO1 transfection which including Cd68, Cd48, Itgax, Cd3g, Ncr1, Itgam, and Ly6c, while only three markers of immune cells involving Cd68, Itgam, and Ly6c can be enriched by FoxO3a transfection (141). A recent study also indicated that triggering of FoxO localization by 9-hydroxy-octadecadienoic acid mediated JNK signaling pathway is accompanied with antagonism of insulin signaling, providing a novel linking between dietary fatty acid balance and meta-inflammation (142). In this regard, we assume that inhibition of inflammatory pathways *via* FoxO transcription factors may increasingly be recognized as exciting prospects for the prevention of multiple diabetic disorders.

## Challenges and considerations of Forkhead box in diabetic cardiomyopathy

**FIGURE 4:**
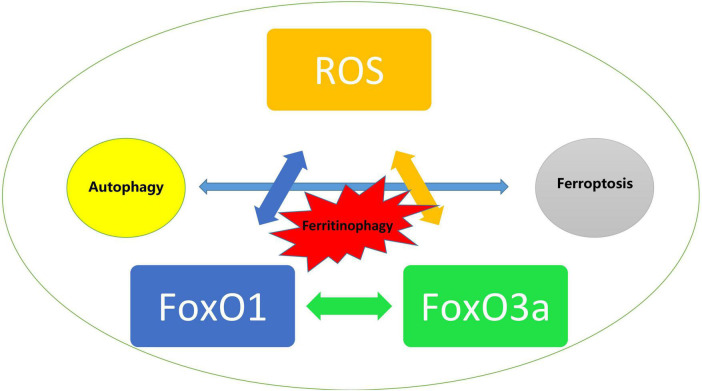
Trigger of ROS mediated activation of FoxO and the impact on autophagy and ferroptosis in diabetic cardiomyopathy.

Despite multiple studies have provided a variety of potential mechanisms acting on DCM and support a dual role of FoxO transcript factors in DCM presumably dependent on the level of oxidative stress, it is noticeable that the participation of oxidative stress possibly proceeds the development of certain signaling transduction and pathophysiological changes including most types of cell death, especially in modulating the relationship between autophagy and ferroptosis that are both targets for FoxO regulation. Ferritinophagy, an unique form of selective autophagy of ferritin has been proved to contribute significantly in the development of cardiovascular diseases (143). Li and colleagues reported that enhanced ferroptosis and activated ubiquitin specific peptidase-19/beclin1-mediated autophagy were observed in oxygen–glucose deprivation/reoxygenation-induced H9c2 cell death, which could be reversed by resveratrol treatment *via* attenuating the oxidative stress injury (144). Given that ROS are also associated with other cell death pathways, and that FoxO is recognized as playing an dual role in transcriptional regulation of proteins in autophagy as we described previously, we assume that FoxO may impact on ferroptosis in both autophagy dependent and independent manners *via* ROS as the trigger during the development of DCM. On the other hand, FoxO1 and FoxO3a exerted the same protective effects in H9C2 cells under hyperglycemia as we investigated before (16) and the deficiency of either of them leads to the identical consequences that aggravates meniscus lesions in experimental models through abolishing autophagy and antioxidant defense genes (145), whereas FoxO1 or FoxO3a may have radically different effects in diabetic hearts and they even interfere with each other. For instance, FoxO3a may increase autophagy that is mediated and associated with the promotion of the translocation of FoxO1 from the nucleus to the cytoplasm, which suggested that the process dominated by FoxO3a cannot be separated from FoxO1 (146). However, it is yet hard to know whether FoxO1 or FoxO3a may play a leading role in the development of DCM (Figure 4).

## FoxO1 and FoxO3a as potential therapeutic target of diabetic cardiomyopathy and possible effects of the natural product salvianolic acids

In the above sections, we reviewed the recent progress on FoxO multiple post-translational modifications and mechanisms, which involve various signal transduction pathways in DCM. In the meanwhile, it should be noted that currently pharmacological therapies that can specifically and effectively target FoxOs in the clinical settings are lacking. Interestingly, we noticed that salvianolic acids (mainly refer to hydrophilic constituents of salvianolic A and B) extracted from the roots of a traditional Chinese medicinal herb, *Salvia miltiorrhiza*, have been emerged as potent antioxidants that confer protection against cardiovascular disease (147, 148). For instance, several studies demonstrated salvianolic acid A conferred cardio-protective effects as a free radical scavenger through targeting the Akt/GSK-3β, JNK, PI3K/Akt, and ERK1/2 signaling pathways, and reduced myocardial ischemia/reperfusion injury in diabetic rats and cellular hypoxia/reoxygenation injury in *in vitro* models that involved the reduction cardiomyocytes apoptosis (149–151). Similar to salvianolic acid A, salvianolic acid B can also act on the PI3K/Akt signaling pathway and the ERK1/2 signaling pathway, both of which are closely associated with myocardial damage (152, 153). Furthermore, cardiac dysfunction and fibrosis in the heart of diabetic rats could be ameliorated by salvianolic acid B through down-regulating of insulin-like growth factor-binding protein 3 (IGFBP3) activity by enhancing myocardial angiogenesis (154). The above-mentioned cardio protective effects of salvianolic acids might stem from their unique polyphenolic structure (135, 155, 156). Additionally, salvianolic acid A treatment yield distinct protective effects against cerebral ischemia/reperfusion injury *via* inhibition of the FoxO3a/BIM pathway both *in vivo* and *in vitro* (157), whilst salvianolic acid B could mitigate tunicamycin-mediated cell pyroptosis *via* modulating of AMPK/FoxO4 and Syndecan-4/Rac1 signaling pathways (158). Salvianolic Acid B has also been shown to decrease oxidative stress reaction by regulating SIRT3/FoxO1 signaling pathway and play a therapeutic role in the treatment of non-alcoholic steatohepatitis in rats (159). The above findings collectively suggest that salvianolic acids may serve as a potential drug candidates in cardiovascular disease therapy in general and in DCM in particular through targeting FoxOs directly or indirectly *via* their strong ROS scavenging properties (160, 161), given that FoxO represent viable target of DCM, although relevant and existing investigations are rare. Further in depth studies are merited to figure out explicit mechanisms between FoxO and salvianolic acids in the prevention and/or treatment of DCM in view that these natural products are safe and do not have major side effects at the doses used to treat cardiovascular diseases (162).

## Conclusion

In the current review, we discussed mechanisms and pathways that are disturbed in the diabetic heart and structure as well as regulation of FoxO in DCM. However, above discussions mainly based on animal and cell experiments instead of real clinical applications while the integrated and precise signaling of diabetic cardiac injury or the relate contribution of FoxO transcription factors in DCM remain largely unknown. In order to seize the target spot of FoxO in DCM, it is necessary to determinate whether the observed changes is available and adaptive both in TID and T2D, while oxidative stress is widely accepted as a promising pointcut under high glucose condition. Meanwhile, given the interplay between different mechanisms, it is also important to achieve a comprehensive confirmation of many of these molecular underpinnings that regulate and coordinate these processes to attenuate injury in the human diabetic heart to gain more insight if potential treatment targets identified in animal models may also be a valid target in humans.

## Author contributions

RH, HH, WX, JL, HL, JT, and ZX: conceptualization, literature review, tables, writing – review and editing, revisions, and final editing. All authors have read and agreed to the published version of the manuscript.

## Abbreviations

DCM: diabetic cardiomyopathy
FoxO: the class O of Forkhead box
T2D: type 2 diabetes
T1D: type 1 diabetes
PDK4: pyruvate dehydrogenase kinase 4
Akt: protein kinase B
NLRP3: NOD-like receptor family protein-3
SGK: serum and glucocorticoid-regulated protein kinase
CK1: casein kinase-1
PI3K: phosphatidylinositide3-kinases
PDK1: 3-phosphoinositide-dependent protein kinase-1
SIRT1: silent information regulator
Mdm2: murine double minute-2
ERK: extracellular signal-regulated kinase
ATP: adenosine triphosphate
ROS: reactive oxygen species
Ang II: Angiotensin II
SIRT3: sirtuin-3
IL: Interleukin
TXNIP: thioredoxin-interacting protein
Nrf2: nuclear factor erythroid2-related factor 2.
